# A Rare Incidental Finding of Phrygian Cap in a Case of Pyloric Perforation

**DOI:** 10.7759/cureus.32451

**Published:** 2022-12-12

**Authors:** Varun Kulkarni, Harshal Ramteke, Yashwant Lamture, Pankaj Gharde, Tushar Nagtode, Venkatesh Rewale

**Affiliations:** 1 Department of General Surgery, Jawaharlal Nehru Medical College, Datta Meghe Institute of Medical Sciences, Wardha, IND

**Keywords:** duplication of gall bladder, septate gall bladder, anomalous gall bladder, liberty cap, gall bladder

## Abstract

Deviation from the normal development of the biliary tree and gall bladder gives rise to numerous different types of anomalies. One of the anomalies is the Phrygian cap. The Phrygian cap is an even rarer condition. We report a case that was asymptomatic. In a rare case, the Phrygian cap anomaly may cause symptoms that are mainly due to superadded cholelithiasis. A detailed pre-operative history and thorough physical examination should be done. Many of the cases may be missed during the routine examination. The diagnosis, with the help of radiological investigations, will be helpful to keep the location and anatomy of the anomalous gall bladder in mind during the intra-operative period. The patient was operated on to seal off a perforation, where a Phrygian cap was observed as an incidental finding. The patient did not have any complaints pointing toward the presence of cholelithiasis or cholecystitis. Due to the asymptomatic incidental presentation of the condition, the operating surgeons decided on avoiding further dissection and an increase in the morbidity load. Thus, we report this case to focus on this rare entity and consider it a differential while dealing with a case of pain in the abdomen.

## Introduction

Numerous anomalous deviations from the normal development of the gall bladder have been documented. The symptoms and their severity may vary. These conditions should be kept in mind while dealing with cases of pain in the abdomen, especially in the right hypochondrium. The Phrygian cap is an infrequently encountered congenital anomaly [1]. As the condition is most commonly asymptomatic and is usually an incidental finding, the anomaly of the gall bladder is not widely available in the literature [2]. In cases of an incidental finding, it depends on the operative surgeon, who might not opine in favour of operating on an asymptomatic gall bladder [2].

Early diagnosis of the condition is crucial to decreasing morbidity and mortality. A detailed preoperative history and a thorough physical examination should be done. The diagnosis, with the help of radiological investigations, will be helpful to keep the location and anatomy of the anomalous gall bladder in mind during the intra-operative period. This helps in avoiding intra-operative complications.

The differentials to be kept in mind while dealing with a case of duplication of the gall bladder are diverticula of the gall bladder, fold of the gall bladder, Phrygian cap, choledochal cyst, pericholecystic fluid, etc. [3]. In a few cases mentioned in the literature, a re-operation was summoned due to the development of cholecystitis in the remaining gall bladder [4]. The anomalous nature of the gall bladder can be asymptomatic in many cases. Rarely, recurrent abdominal pain can be a result of gallstones due to an underlying septate gall bladder [5]. According to the literature, the condition of having a simple septum is more common than cases with multiple septae. Post-inflammatory formation of adhesions and compartmentalization of the gall bladder cavity have also been described [6]. We report a case where the anomaly was suspected on imaging studies and confirmed intra-operatively.

## Case presentation

**Figure 1:**
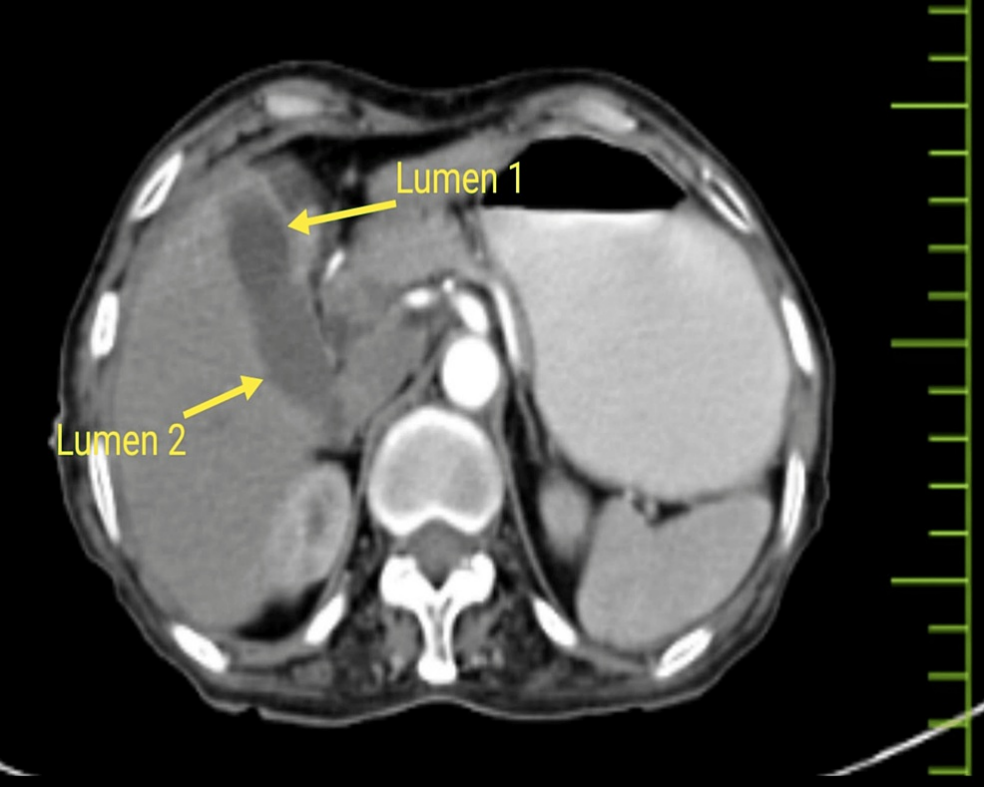
Computed tomography showing abnormally shaped gall bladder

**Figure 2:**
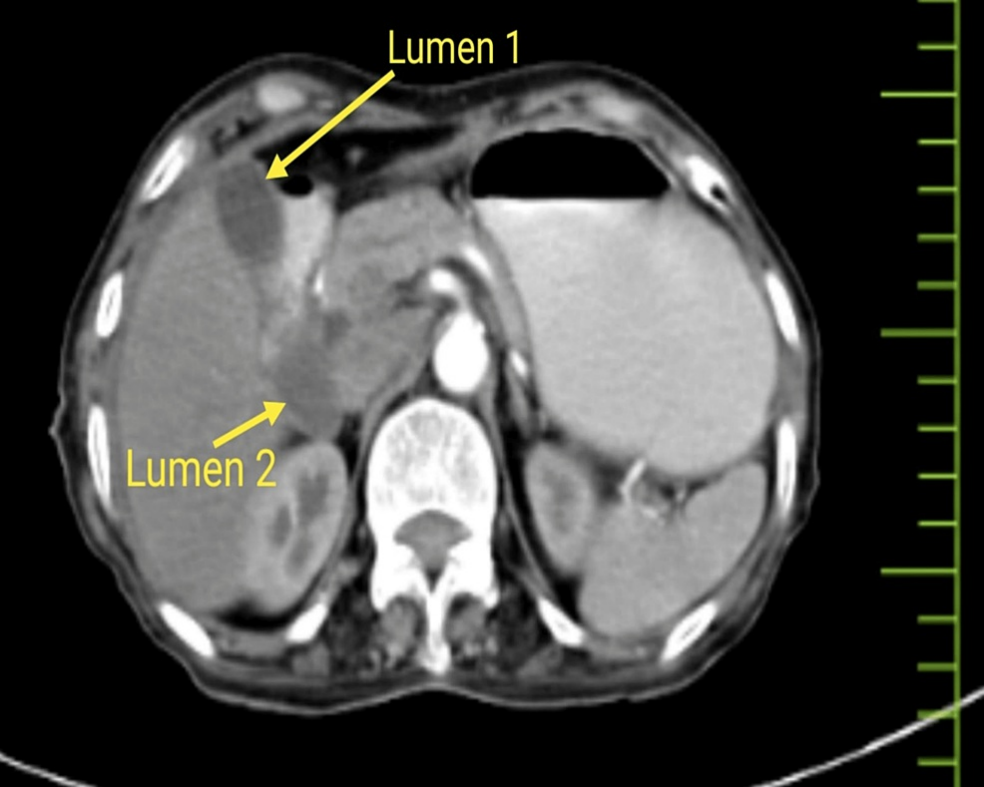
Computed tomography showing anomalous gallbladder

A 65-year-old female presented to our emergency department with complaints of pain in the abdomen for 15 days. On palpation, the patient had mild tenderness in the epigastric region, and the vitals were normal. All routine blood investigations were done and were within normal limits. The X-ray abdomen was erect, and contrast-enhanced computed tomography revealed pneumoperitoneum and a suspected anomaly of the gall bladder (Figures 1-2).

**Figure 3:**
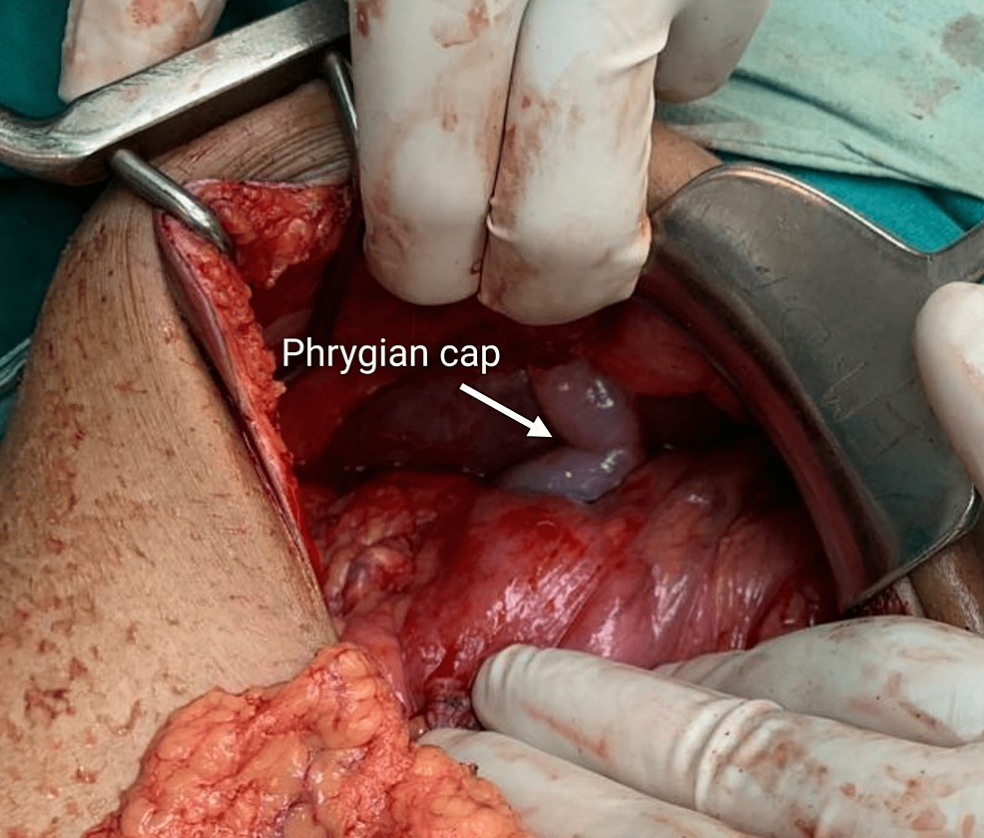
Intra-operative finding of abnormal gallbladder

**Figure 4:**
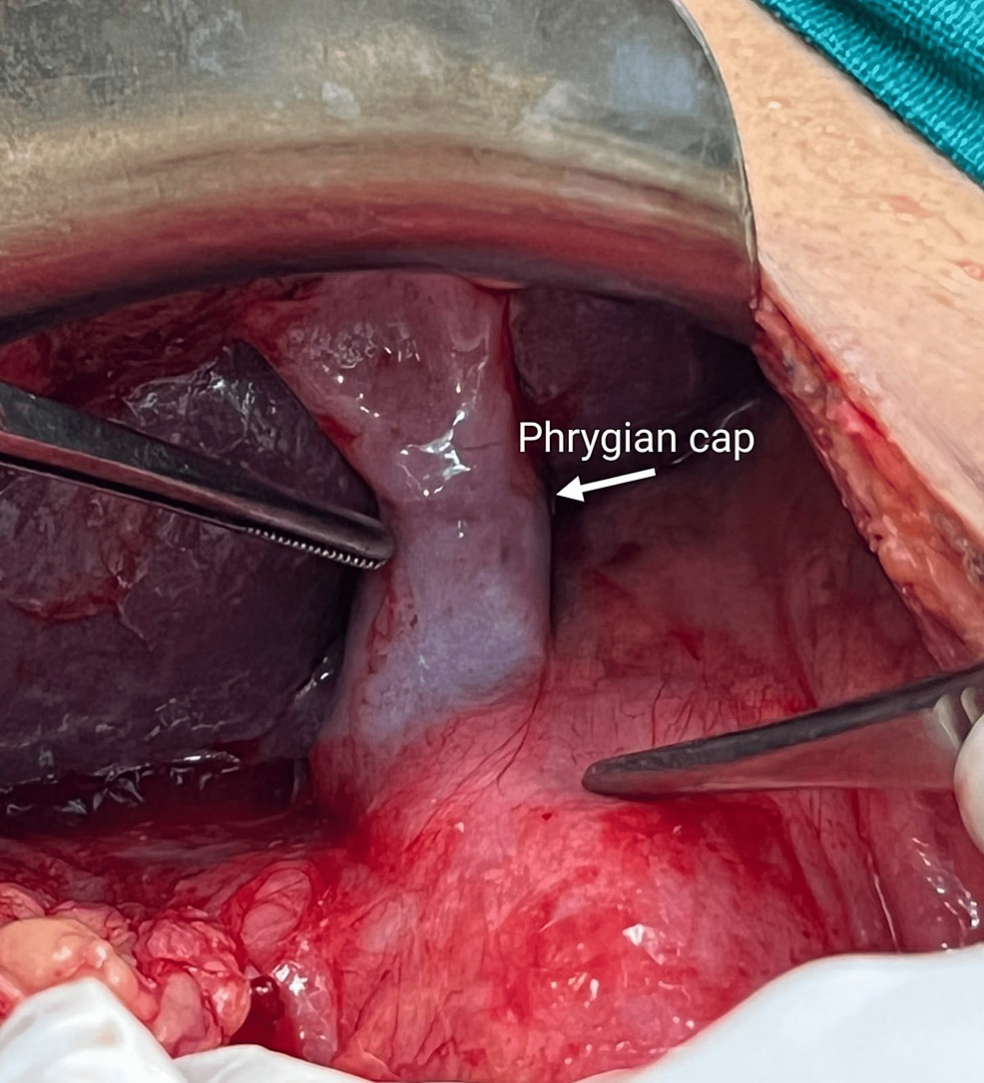
Phrygian cap

Thus, the patient was taken for emergency surgery. The emergency exploratory laparotomy revealed a sealed-off perforation at the pyloric part of the stomach. The abdomen was examined, and there was no evidence of any other perforation or intestinal pathology. The visible abdominal viscera were normal. The gall bladder region was dissected carefully until the Calot’s triangle was visualized. The surgeons observed a single artery and a single cystic duct without any evidence of duplication of the gall bladder. The diagnosis of a rare anomaly of the gall bladder, namely the ‘Phrygian cap’, was confirmed (Figures 3-4).

As the anomaly was asymptomatic, the operating surgeons avoided further dissection in this case. The perforation at the pyloric part of the stomach was a sealed-off perforation, and Graham's patch repair was done by the surgeons. Post-operatively, cancer antigen (CA) 19.9 and alpha-fetoprotein were sent, which came back within the normal limits. The core needle biopsy from the gall bladder wall was negative for any evidence of malignant cells. Post-operatively, the patient was stable and was discharged with advice to follow up at a later date.

## Discussion

The autopsy reports of about 2-6% of the cases show the congenital abnormality famously known as the Phrygian cap, which is mentioned as a rare abnormality in the literature [7]. It resembles the caps of the ancient Phrygian people of Anatolia (modern-day Turkey), the liberty variety of caps of the French Revolution, or the caps of the Smurfs. The abnormality occurs because the gallbladder fundus has a fold on itself. One of the commonest congenital anomalies of the hepatobiliary tree is a folded type of fundus of the gall bladder. This abnormality was described as a 'Phrygian cap' deformity because of its resemblance to the bonnet worn by the Phrygian community of Asia. This was mentioned in the literature by Boyden in his study performed in the early twentieth century [8].

In our case, there was a sealed-off perforation at the pyloric end of the stomach and an incidental finding of a Phrygian cap. However, the pre-operative diagnosis of the anomaly helps decrease possible morbidity and mortality. Various investigation modalities such as ultrasonography, oral cholecystography (OCG), endoscopic retrograde cholangiopancreatography (ERCP), computed tomography scan, and magnetic resonance imaging (MRI) are performed for diagnosing a Phrygian cap type of anomaly. ERCP is an invasive kind of method, thus it is seldom advised. The most commonly advised method of investigation is an ultrasonographic examination, though, in confused patients, CT and MRI scans can also be preferred for making out the anatomical structure of the hepatobiliary tree. Prophylactic cholecystectomy is not usually recommended in every case of an abnormal gall bladder [9]. In symptomatic cases associated with deformity, surgical intervention is advised despite the absence of gallstones [10]. A thorough pre-operative anatomical evaluation should be done in detail for such patients to eliminate intra-operative duct system injuries. The laparoscopic approach is the gold standard, as per many authorities, as it is a safe method for the removal of double gallbladders. This same approach can also be advised for a case of the Phrygian cap if the condition was diagnosed pre-operatively and the patient is symptomatic [10]. The term was first coined by Bartel et al. in their studies on autopsy cases, which were further popularised by Boyden et al. [11,12]. The literature has suggested a Phrygian cap predisposing to cholelithiasis, but there is no absolute confirmatory documentation [13].

## Conclusions

With this case, we would like to bring the attention of our fellow surgeons toward this rare entity, which should always be kept in mind as a differential while dealing with cases presenting with complaints consistent with gall bladder diseases. Early and pre-operative diagnosis of the condition helps decrease the morbidity and mortality load on society; thus, a proper medical history and a thorough physical examination are very important. Radiological modalities are of utmost importance as the condition may be silent, as in our case, and can precipitate intra-operative complications if missed pre-operatively.
